# Primary Spontaneous Pneumothorax in a 32-Week Complicated Pregnancy

**DOI:** 10.7759/cureus.15037

**Published:** 2021-05-15

**Authors:** Nikolaos Georgopapadakos, Dimitrios Lioumpas, Georgios Mpenakis, Alexios Tsochrinis, Ermioni Tsarna

**Affiliations:** 1 Obstetrics and Gynecology, General Hospital of Nikaia “Agios Panteleimon”, Piraeus, GRC; 2 Thoracic Surgery, General Hospital of Nikaia “Agios Panteleimon”, Piraeus, GRC

**Keywords:** pneumothorax, spontaneous, pregnancy, video-assisted thoracoscopic surgery (vats), ultrasound chest

## Abstract

Primary spontaneous pneumothorax during pregnancy is a very rare entity. We present a 37-year-old Caucasian woman with spontaneous pneumothorax during the 32nd week of her fourth pregnancy who was treated with intercostal chest drain and was followed up with chest ultrasound. The patient experienced two more episodes of recurrent pneumothorax during pregnancy and puerperium and a uniportal video-assisted thoracoscopic surgery (VATS) was performed. Few such cases have been reported previously in the literature, and there are no relevant medical guidelines. Treatment options include surveillance of a collapsed lung, conservative management with intercostal chest drain, and surgical management with the use of VATS.

## Introduction

During pregnancy, and especially during the third trimester, many changes occur with regard both to anatomy and physiology of the human body. The uterus becomes eight times larger than in a non-pregnant woman, while pregnancy hormones elicit a systemic effect. As a result, a sequel of adaptations takes place, ultimately affecting the respiratory system. Relevant anatomical adaptations include the elevation of the diaphragm, the decrease of functional residual capacity and expiratory reserve volume, and the increase of all chest dimensions, apart from its height, which results in a barrel-shaped chest [1]. With regard to hormonal changes, the increase of progesterone levels causes smooth muscles relaxation, thus inducing bronchodilation [1]. In addition, progesterone levels affect the respiratory center in medulla oblongata by increasing its sensitivity to carbon dioxide [1]. Finally, the amount of air that a woman breaths in and out increases as the pregnancy progresses, as does the oxygen consumption [1]. Because of the aforementioned changes, many women feel shortness of breath or report a subjective feeling of breathing difficulty, complaints that usually worsen in the third trimester of pregnancy. As anticipated, in case of a pre-existing respiratory condition or when lung diseases develop during pregnancy, respiratory compensation might be difficult to achieve.

Primary spontaneous pneumothorax (PSP) is defined as the presence of air in the pleural cavity, in the absence of trauma [2]. The rupture of a bleb or bulla is usually regarded as its cause [2]. Patients who experience PSP are typically tall and thin. Risk factors further include male sex and smoking, while atmospheric pressure changes and exposure to loud music are regarded as triggering factors [2]. The reported incidence of PSP is estimated to affect 7.4-18 males and 1.2-6 females per 100,000 population [2]. Symptoms usually include sudden ipsilateral chest pain and shortness of breath [2]. On rare occasions, spontaneous tension pneumothorax may develop, resulting in a life-threatening condition. Thus, accurate and quick diagnosis of PSP in pregnant women is crucial, as both maternal and fetal life are at risk.

## Case presentation

**Figure 1:**
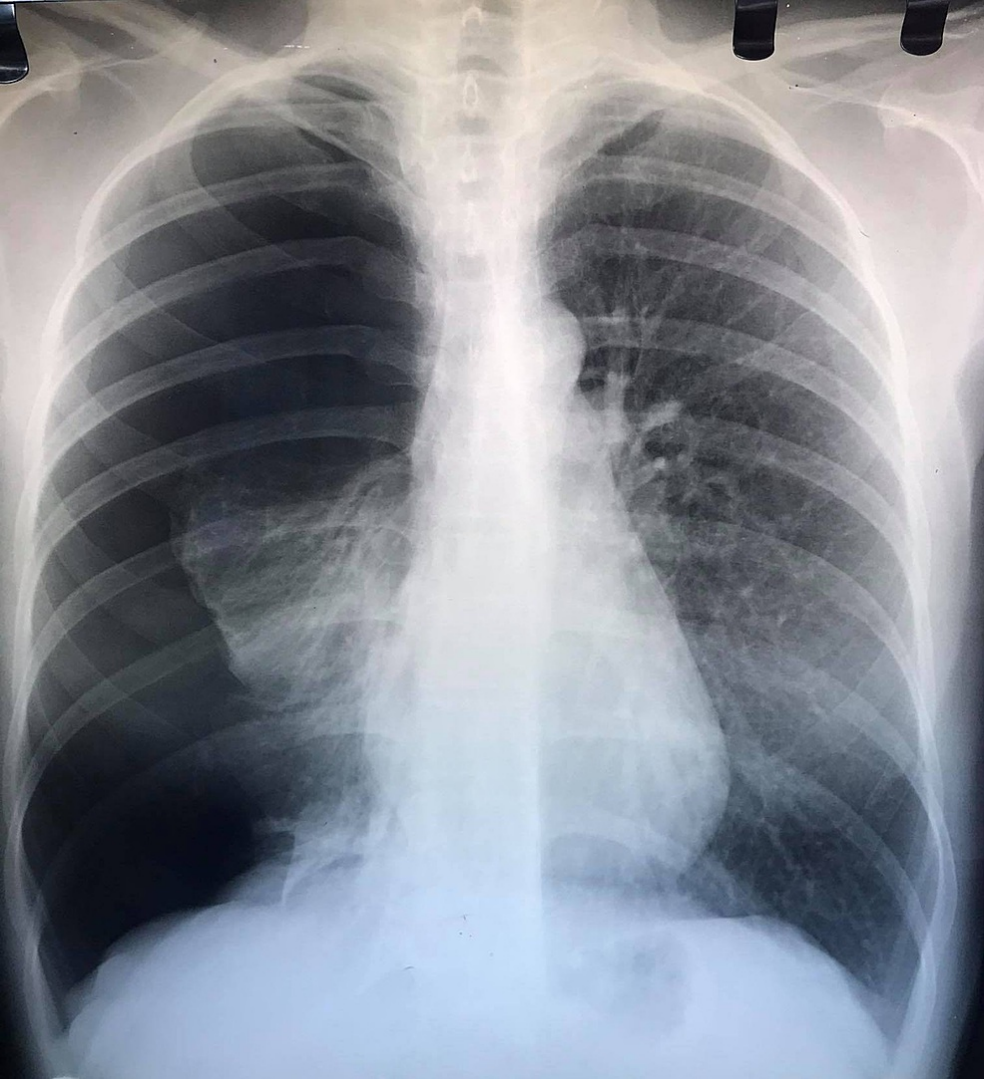
Chest X-ray showing a right pneumothorax with the right lung completely collapsed

**Figure 2:**
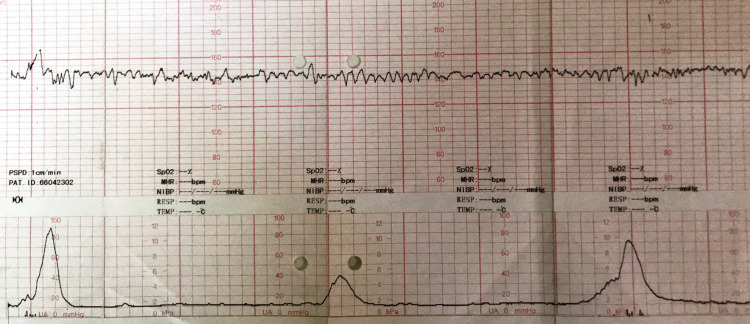
Reactive non-stress test showing myometrial contractions

We present a 37-year-old Caucasian woman at 32 weeks of pregnancy reporting shortness of breath and chest pain; the symptoms had sudden onset while patient was at rest. She was a non-smoker, with no history of pulmonary disease. Her body mass index prior to pregnancy was 28 and she had conceived naturally. She was gravity 4 parity 3, had three normal labors, and no pregnancy loss. No allergies or surgery were reported in her personal medical history. Regarding medications, she was currently receiving iron, calcium, and magnesium supplements because of her pregnancy. On physical examination, patient was severely tachypnoeic (respiratory rate 38) with absence of auscultation sounds of the right lung and a hyperresonant percussion sound over the right hemithorax. Oxygen saturation was 93% on air, while blood gas analysis revealed borderline arterial oxygen and carbon dioxide partial pressure values. Chest X-ray revealed a completely collapsed right lung, a finding that was confirmed by lung ultrasound (U/S) (Figure 1). Severe acute respiratory syndrome coronavirus 2 rapid test and polymerase chain reaction test were negative. Full blood count and inflammation markers were within normal range (Table 1). Biochemical blood analyses were also normal, with the only exception of the enzyme creatine kinase that was 242 IU/ml (normal laboratory range 26-192 IU/ml) (Table 1). An abdominal U/S was performed without any pathological findings. In addition to the aforementioned symptoms, patient reported mild abdominal cramps, as well as abdominal and lower pelvic pressure. The non-stress test (NST) revealed myometrial contractions (Figure 2), and the intense pain due to PSP was regarded as their cause. Bishop score was less than 2, cervical dilatation was 1 cm, and cervical length was estimated by transvaginal U/S to be 30 mm.

**Table 1:** Full blood count and biochemical analyses results from a 37-year-old Caucasian woman with primary spontaneous pneumothorax at 32 weeks of pregnancy

Blood test	Result
White blood cells	7.900 /μl
Neutrophils	78.3%
Lymphocytes	17.7%
Monocytes	3.2%
Red blood cells	3.46 M/μl
Hemoglobin	11.4 g/dl
Hematocrit	33.0%
Platelets	138,000/μl
Glucose	102 mg/dl
Blood urea nitrogen	16 mg/dl
Creatinine	0.6 mg/dl
Na	138 mmol/l
K	3.4 mmol/l
Creatine kinase	242 IU/l
Lactate dehydrogenase	214 IU/l
Aspartate aminotransferase	17 IU/l
Alanine aminotransferase	13 IU/l
Alkaline phosphatase	71 IU/l
Gamma-glutamyl transferase	13 IU/l
C-reactive protein	1.9 mg/l

**Figure 3:**
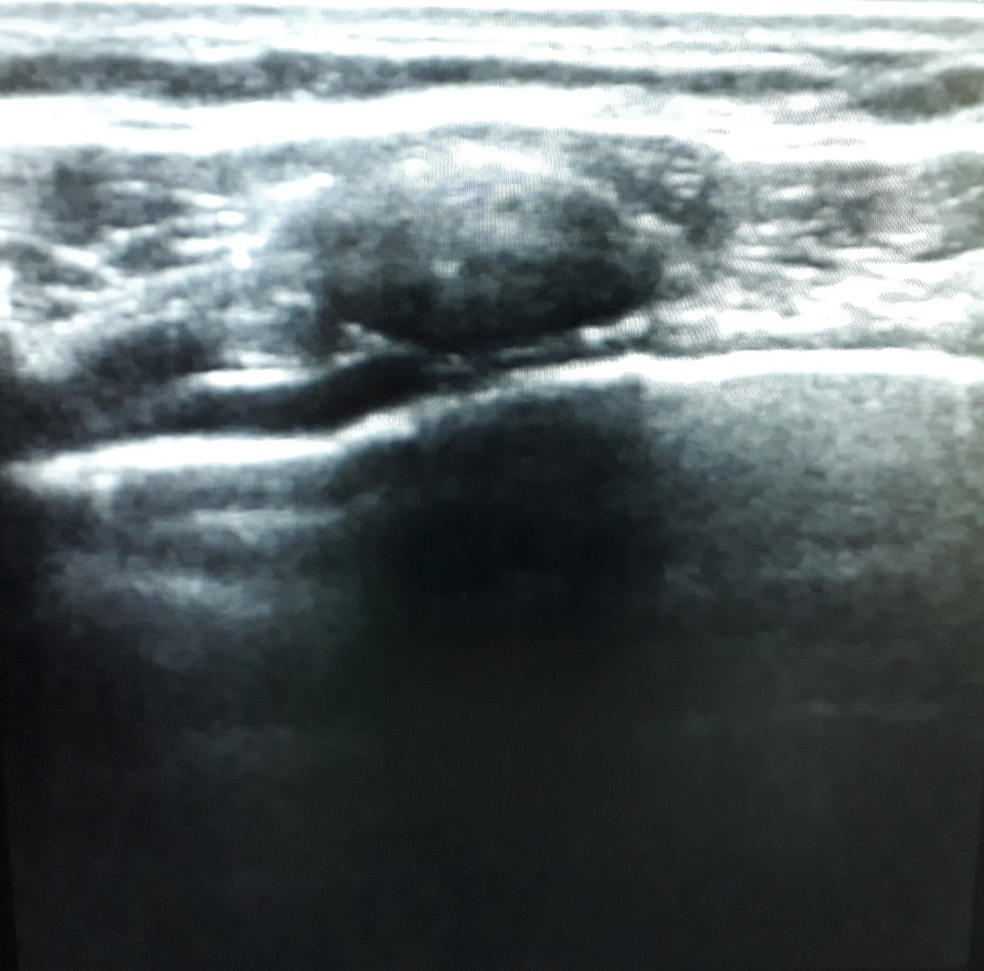
Lung ultrasound image showing minimal amount of air between the lung parenchyma and the chest wall

**Figure 4:**
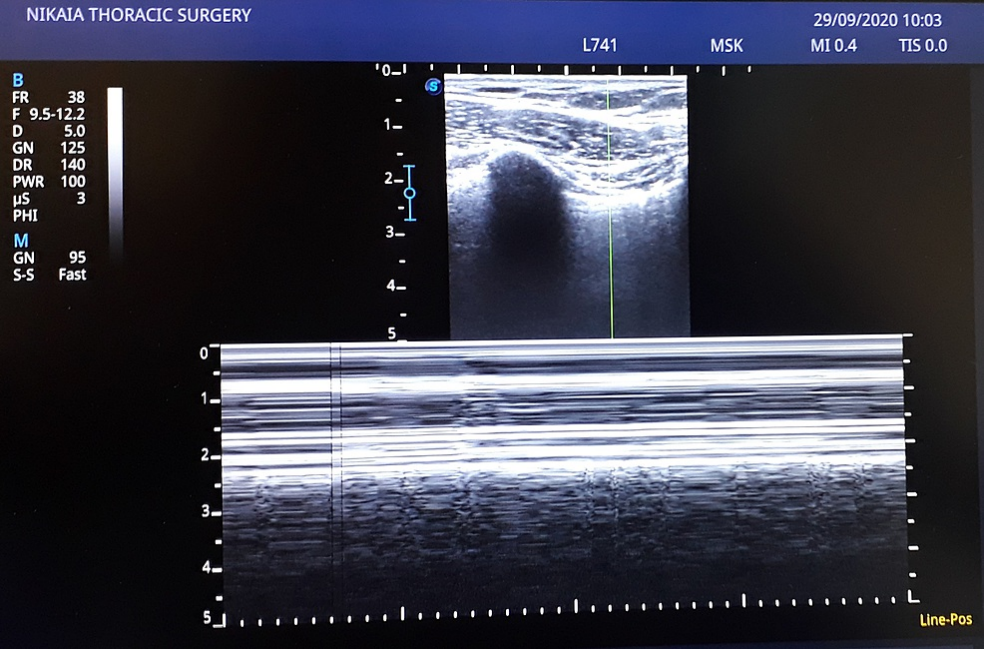
M-mode image of the right lung with the sand-on-the-beach sign, indicating normal motion of the lung during inhalation and exhalation

Based on the diagnosis of PSP and threatened preterm labor, the patient was admitted in the obstetrics and gynecology department. Pneumothorax was treated with the insertion of a 22F intercostal tube drainage (Billau) by a thoracic surgery specialist, and it was closely monitored daily with the use of bedside lung U/S. Intravenous paracetamol (3 g/day) plus additional use of small doses of pethidine (25-100 mg intramuscularly [IM]) were used as analgesic protocol treatment. To repress labor and avoid neonatal prematurity, atosiban (oxytocin inhibitor) was administered intravenously as a tocolytic. Furthermore, betamethasone was administered (two doses of 12 mg IM, 24 hours apart) with the aim to aid fetal lungs maturation, in case that preterm birth could not be avoided. After one day of continuous atosiban administration, the uterine contractions stopped and the NST recorded only the basal myometrial tone. Fetal wellbeing was ensured based both on record of fetal heart rate at the NST and normal Doppler examination via transabdominal U/S. After one week of hospitalization, the intercostal drainage (Billau) was removed as the pneumothorax had resolved (Figure 3 and Figure 4). The patient was, therefore, discharged from the hospital under close medical attention.

At 37 weeks of gestation the patient reported once again chest pain and lung U/S confirmed a recurrent PSP. Once again, an intercostal tube drainage (Billau) was placed. The obstetric evaluation revealed cervical dilation of 3 cm and uterine contractions with maximum peak pressure of 60 mmHg were recorded at the NST. Despite our recommendations for vaginal labor and safety assurance, the patient opted for a cesarean section. The baby was born in good general condition and weighing 3160 g.

**Figure 5:**
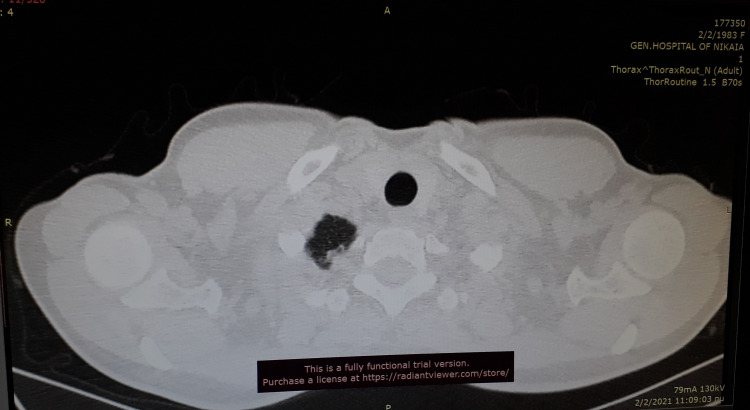
The chest CT scan showing small apical blebs at the right superior lobe

**Figure 6:**
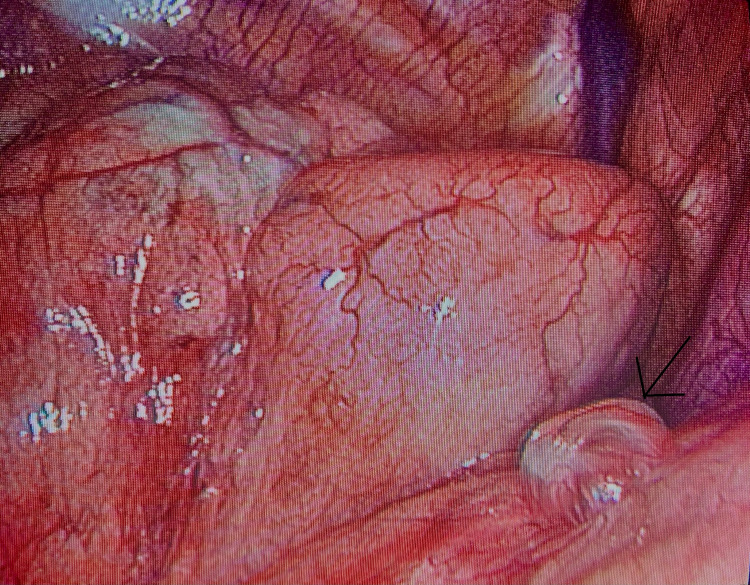
Image obtained during uniportal video-assisted thoracoscopy showing the blebs (arrow)

During the sixth week of puerperium, the patient experienced a third episode of PSP with similar symptoms, although dyspnea was less severe compared to the previous episodes during pregnancy. After emergency intercostal tube drainage (Billau) placement and pneumothorax resolvement, a high-resolution chest CT scan (slice thickness of 1.5 mm) was performed, revealing the presence of small apical blebs (Figure 5). A uniportal video-assisted thoracoscopic surgery (VATS) was performed with excision of the air-filled pulmonary blebs and apical pleurectomy (Figure 6). Multiple biopsies were obtained, which revealed emphysema-related lesions of the lung with no further findings. The patient’s follow-up was uneventful and she remained free of symptoms three months after surgery.

## Discussion

PSP during pregnancy is a relatively rare entity, although its exact incidence is unknown and might actually be underestimated in cases of partially collapsed lung or mild clinical symptoms [3]. Nonetheless, less than 100 cases have been reported in the literature, and medical guidelines to aid clinical management are totally lacking [3]. PSP usually affects young pregnant women below 30 years of age, during the second and third trimesters of pregnancy [3]. It is, however, a potentially serious condition for both the pregnant woman and her fetus. Taking into consideration that a pregnant woman has 20% increased oxygen demands during pregnancy and 50% increased oxygen demands during labor, failure to achieve respiratory compensation may additionally lead to severe fetal hypoxia [4].

During pregnancy, patient’s follow-up for PSP should minimize the fetal exposure to any kind of radiation. Thus, even though CT scan is regarded the reference standard, it should not be performed unless it is deemed absolutely necessary for ensuring the pregnant woman’s wellbeing. In this case, abdomen should be shielded. Similarly, chest X-rays should not be performed routinely. On the contrary, chest U/S has been shown to be both safe for the fetus and has higher sensitivity in the diagnosis of PSP when compared to chest X-ray [5]. Most U/S devices are nowadays portable, providing clinicians with a bedside accurate and instant diagnosis. In addition, the need for patient’s transport is obviated, which is especially helpful in critically ill and hemodynamically unstable patients.

Regarding patient’s management in the case of PSP during pregnancy, first-line treatment options include surveillance with medical management and chest tube drainage (Billau) [6]. In case of inability of lung re-expansion or recurrent episodes of PSP, VATS is recommended. Other therapeutic options that were widely used in the past, such as plurodesis, sclerotherapy, laser treatment, resection of blebs or pleura, and open thoracotomy, have been largely replaced by VATS. Notably, prior to any invasive therapeutic intervention before the completion of the 36th week of pregnancy, tocolytic drugs should be administered.

Vaginal delivery is not contraindicated in case of a successfully treated PSP during pregnancy, but other maternal or fetal indications should be considered. Notably, hyperventilation and Valsalva maneuver during labor are thought to increase the risk for recurrent PSP [3]. Therefore, the obstetric team should be prepared for this event and a thoracic surgeon should be readily available. Naturally, the operation theater should be also informed beforehand, in case that an emergency cesarean section is needed for maternal respiratory compromise and/or fetal hypoxia. Based on the reported cases in the literature, obstetric outcome is generally favorable and fetal complications are rare [3]. However, publication bias may be heavily affecting the reported outcomes, since all data arise from single-case reports rather than cohort studies or consecutive case series and unfavorable outcomes are less likely to be reported in the literature.

## Conclusions

PSP during pregnancy can be treated in a similar manner as in non-pregnant women, even though specific medical guidelines do not exist. Prognosis is generally good both for the mother and the baby. Chest U/S seems to be the best method for diagnosis and follow-up of pregnant women with PSP. The majority of pregnant patients with PSP are initially treated conservatively with a chest tube; tocolytics should be administered before tube’s insertion. VATS should be used in case of recurrent PSP episodes or failure of conservative treatment.
